# Is the maternal health voucher scheme associated with increasing routine immunization coverage? Experience from Bangladesh

**DOI:** 10.3389/fpubh.2023.963162

**Published:** 2023-02-02

**Authors:** Nazia Sultana, Aazia Hossain, Hemel Das, Saseendran Pallikadavath, Mira Koeryaman, Mohammad Rahman, Asiful Haidar Chowdhury, Abbas Bhuiya, Shehrin Shaila Mahmood, S. M. A. Hanifi

**Affiliations:** ^1^Health Systems and Population Studies Division, International Centre for Diarrhoeal Disease Research, Bangladesh (icddr, b), Dhaka, Bangladesh; ^2^Portsmouth-Brawijaya Centre for Global Health, Population, and Policy, University of Portsmouth, Portsmouth, United Kingdom; ^3^Salford Business School, University of Salford, Salford, United Kingdom; ^4^ARK Foundation, Dhaka, Bangladesh

**Keywords:** immunization, antenatal care, Bangladesh, demand side financing, maternal health voucher scheme

## Abstract

Bangladesh initiated the Maternal Health Voucher Scheme (MHVS) in 2007 to improve maternal and child health practices and bring equity to the mainstream of health systems by reducing financial and institutional barriers. In this study, we investigated whether the MHVS has an association with immunization coverage in a rural area of Bangladesh. Between 30 October 2016 and 15 June 2017, we carried out a cross-sectional survey in two low performing areas in terms of immunization coverage- Chattogram (erstwhile Chittagong division) and Sylhet division of Bangladesh. We calculated the coverage of fully immunized children (FIC) for 1151 children aged 12–23 months of age. We compared the coverage of FIC between children whose mothers enrolled in MHVS and children whose mother did not. We analyzed immunization coverage using crude odds ratio (OR) and adjusted OR (aOR) from binary logistic regression models. The overall coverage of FIC was 86%. Ninety-three percent children whose mothers were MHVS members were fully immunized whereas the percentage was 84% for the children of mothers who were not enrolled in MHVS. Multivariate analysis also shows that FIC coverage was higher for children whose mothers enrolled in MHVS compared to those children whose mothers did not; the aOR was 2.03 (95% confidence interval 1.11–3.71). MHVS provides a window for non-targeted benefits of childhood vaccination. Providing health education to pregnant mothers during prenatal care may motivate them to immunize their children. Programmes targeted for mothers during pregnancy, childbirth and post-natal may further increase utilization of priority health services such as childhood immunization.

## 1. Introduction

Bangladesh has seen substantial development in maternal and child health over the years. Between 2014 and 2017, the percentage of women receiving antenatal care (ANC) from medically trained providers increased from 64 to 82%, deliveries attended by medical personnel increased from 42 to 53% and receiving postnatal care (PNC) checkup from medically trained providers within 2 days of delivery increased from 36 to 52 (1). However, this increase in maternal and child health services varies in terms of geographical location, socioeconomic status (SES) and education.

Bangladesh introduced MHVS in 2007 to improve maternal and child health practices and bring equity to the mainstream of health systems by reducing financial and institutional barriers. The MHVS is considered one of the most critical programmes, in terms of maternal health, in Bangladesh. The services are financed by the Government of Bangladesh, and beneficiaries can avail them, free of cost. Apart from the direct impact of the voucher scheme on mothers during pregnancy, delivery and postnatal period, there can be some indirect benefits since the concept of safe motherhood has been explicitly linked to disease prevention of children. Literature shows that among other factors such as mother's education; socioeconomic status and age; safe motherhood practices, including antenatal visits, delivery through Skilled Birth Attendant (SBA), institutional delivery and postnatal care are associated with increased immunization coverage (2–6).

According to the 2017–2018 Bangladesh Demographic and Health Survey (BDHS), 86% of children aged 12–23 months received all basic vaccinations by age 12 months (1). This coverage has increased by ten percentage points since 2007 (1). However, there is a gap in immunization coverage due to various factors including geographical location, SES of households, mothers' education and sex of children. Sylhet, which has an FIC of 78%, and Chattogram, which has an FIC of 84% (1), are both considered low-performing areas in terms of immunization coverage.

Evidence suggests that various health interventions, including voucher schemes, have both direct and indirect impacts on child immunization in other similar settings like Bangladesh (7–9). However, the impact of MHVS on immunization coverage has rarely been examined in Bangladesh. We conducted a cross-sectional survey in the Chattogram and Sylhet divisions of Bangladesh to assess whether there is any association between full childhood vaccination coverage and being a member of the MHVS programme.

### 1.1. MHVS in Bangladesh

The MHVS is a demand-side financing designed to provide vouchers and cash benefits to disadvantaged pregnant women in Bangladesh to avail services within the scheme, free of cost (2, 10). Initially MHVS piloted in 21 sub-districts, and currently operates in 53 of the 556 sub-districts in Bangladesh (3, 4). The scheme used universal approach for targeting the nine poorest subdistricts, where all pregnant women of parity 1 or 2 (first or second pregnancy), regardless of poverty status, were offered vouchers. Later on, the targeted approach replaced the universal approach and was implemented in an additional 24 sub-districts, where means-testing was used to identify eligible beneficiaries. The means-testing used the following inclusion criteria: the recipient must be a resident of the sub-district; currently pregnant with their first or second child; own < 6,534 square feet of land; have a household income < US$38.50 per month; and lack ownership of other productive assets.

**Figure 1:**
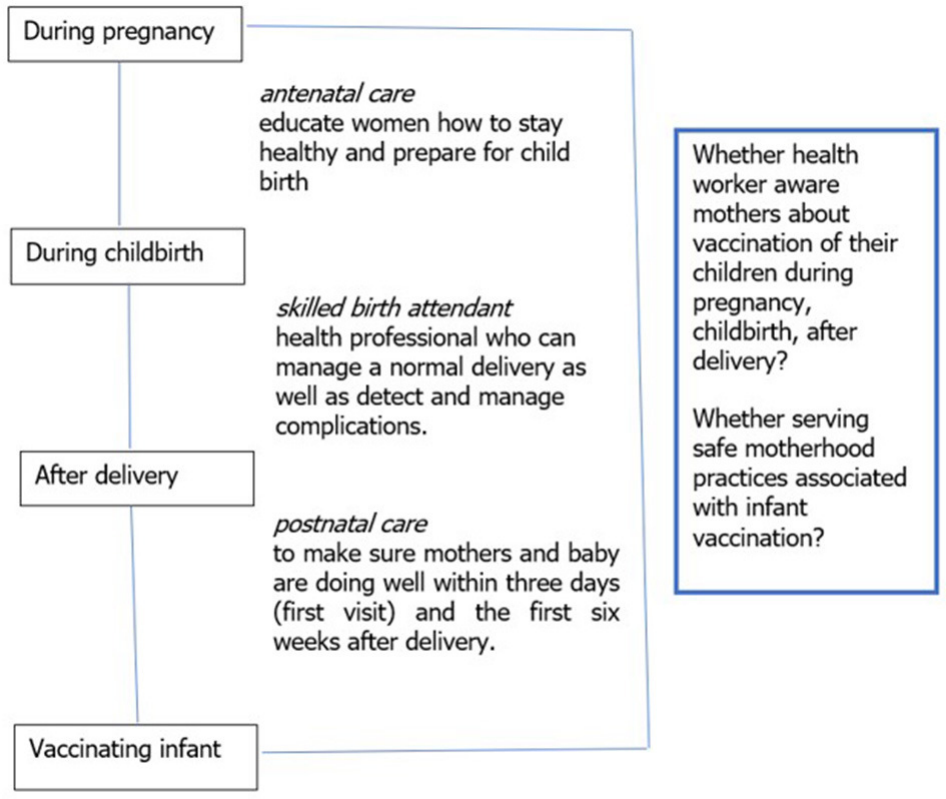
Safe motherhood practices and childhood vaccination.

The scheme covers three ANC visits, delivery at a health facility, one post-natal check-up, free medicines, cash allowances for transport, cash incentive to deliver at a health facility, and management of maternal complications including cesarean delivery, where required (3, 5). MHVS can be used in public and selected private health facilities. For service provision to MHVS beneficiaries, health care providers also receive payment (6). Figure 1 shows current safe motherhood practices and childhood vaccination in Bangladesh.

## 2. Materials and methods

### 2.1. Settings and study population

The study was conducted in two low-performing divisions (in terms of maternal health indicators) of Bangladesh—Chattogram and Sylhet. The areas under Chattogram division are situated in the southeast part of the country including both coastal and hilly regions of the area. The areas under Sylhet division are situated in two settings, *haor* (a wetland ecosystem) and hilly areas. In Chattogram division, we randomly selected two sub-districts Ramu and Teknaf from 11 voucher areas. In Sylhet division, Sreemangal and Sullah sub-districts were randomly selected from five voucher areas (Table 1). Supplementary Figure 1 shows these sub-districts in the map of Bangladesh.

**Table 1:** MHVS sub-districts and sample size, Bangladesh, 2016–17.

**MHVS sub-district**	**Number of women having children 0–23 months**	**Number of mothers having children 12–23 months**
**Chattogram**		
Ramu	600	242
Teknaf	600	254
**Sylhet**		
Sullah	600	215
Sreemangal	600	264
Total	2,400	975

### 2.2. Expanded Programme on immunization (EPI) in Bangladesh

In 1979, The Government of Bangladesh initiated the Expanded Programme on Immunization (EPI) against six preventable diseases: tuberculosis (BCG vaccine); diphtheria, pertussis, and tetanus (DPT vaccine); poliomyelitis (OPV vaccine); and measles (measles vaccine). EPI efforts increased in Bangladesh after 1985, after its commitment to achieve universal child immunization by 1990 (7). Routine vaccination has been one of the most successful programmes to achieve SDG indicator 3.2 which is to reduce neonatal and under-five mortality globally (8) and is considered an effective health intervention for child survival (9). Despite this success, in 2019, globally about 19.7 million children did not receive the three recommended doses of DTP (11).

### 2.3. Definition of variables

#### 2.3.1. Fully immunized children (FIC)

Among children aged 12–23 months who were alive during household visits, those who received BCG, three doses of Penta and OPV, and MV by 12 months of age were considered FIC. The FIC coverage was calculated by dividing the number of children who received the eight vaccination doses before 12 months of age by the number of children visited between 12 and 23 months of age whose vaccination cards were seen at the visit. For children who had more than one visit, vaccination status was determined at the earliest visit at which the vaccination cards were seen.

#### 2.3.2. Antenatal care visit (ANC)

According to the World Health Organization's (WHO) Focused Antenatal Care (FANC) model, one pregnant woman should receive at least 4 ANC during her pregnancy period (12). Aligned with that, we categorized the ANC visit as the following: (a) at least 4 ANC visits and (b) < 4 ANC visits.

### 2.4. Statistical methods

We analyzed immunization coverage using crude FIC coverage ratio (FCR) and adjusted FCR (aFCR) with generalized linear models from the binomial family using the “binreg” command in STATA software. We included sex, mother's age and education, SES, 4+ ANCs, institutional delivery, and receiving care from SBA as potential confounders in the adjusted models.

## 3. Results

### 3.1. Study children

**Figure 2:**
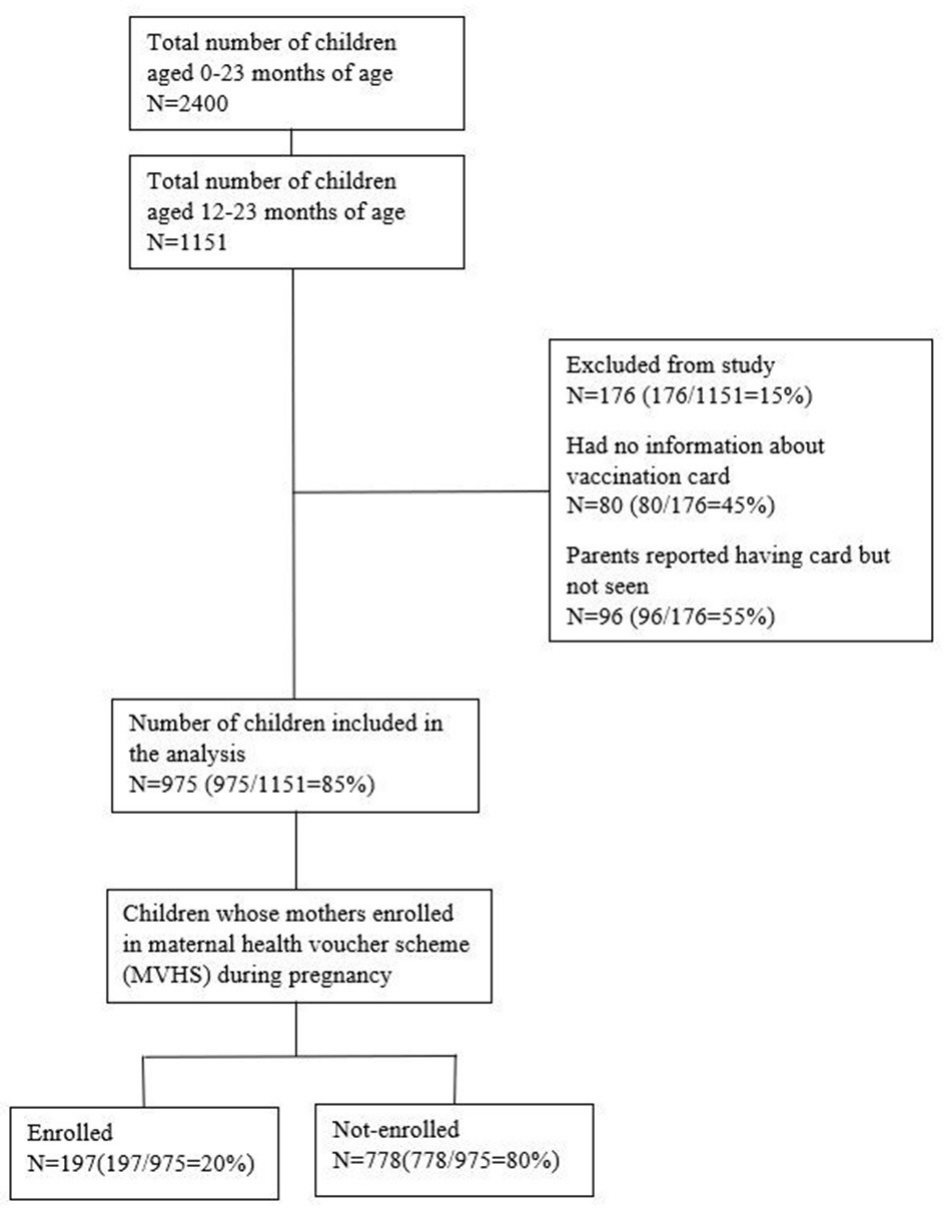
Flow chart of study children.

Between 30 October 2016 and 15 June 2017, information on maternal and child health, demographic, and vaccination details of children from the four MHVS areas were collected from the mothers of children aged 0-23 months through household visits. Six hundred mothers from each of the four MHVS areas were randomly chosen. From the two randomly selected areas in Chattogram, 1,200 mothers were randomly selected from 1,446 eligible candidate mothers. One thousand five hundred two mothers from the two selected MHVS areas of Sylhet fulfilled the eligibility criteria, from which 1200 were randomly selected for interview. Thus, a total of 2,400 women having children < 2 years were interviewed to measure different indicators. As our variable of interest was full immunization coverage of children, we considered the mothers with children aged 12–23 months in this study. Therefore, this study considered 1,151 children whose age ranged from 12 to 23 months. The mothers of these children were interviewed in the survey. A total of 176 children were excluded from the 1,151 children because there was no information on vaccination card or because parents reported having the card but interviewer did not see card during interview. Eventually, 975 children aged 12–23 months were included in the analysis (Figure 2).

Table 2 presents the background characteristics of the study children according to their mothers' membership in MHVS. The table reflects that there is significant difference in background characteristics of members and non-members of MHVS program for all variables with the exception of the sex of the child and whether the beneficiaries are above or below the lower poverty line. It further indicates that receiving four or more ANC (46% for MHVS members vs. 24% for non-members), institutional delivery (members: 57% vs. non-members: 29%), delivery through SBA (members: 53% vs. non-members: 24%) and normal delivery (members: 15% vs. non-members: 9%) were more prevalent among the MHVS members than non-members.

**Table 2:** Background characteristics of study children according to MHVS area.

**Characteristics**	**MHVS area**		***P*-value**
	**Member** **%(*****n*****)**	**Non-member** **%(*****n*****)**	
Number	197	778	
**Age of mother**			
< 25 year	53% (105)	33% (257)	**< 0.001**
25–34 year	45% (88)	55% (425)	
35+ year	2% (4)	12% (96)	
**Mother's education**			
None	18% (35)	30% (231)	**< 0.001**
1–5	36% (71)	40% (308)	
6 and above	46% (91)	31% (239)	
**SES**			
Lowest	16% (32)	22% (168)	**0.029**
Second	19% (38)	24% (186)	
Middle	18% (35)	20% (153)	
Fourth	24% (48)	16% (127)	
Highest	22% (44)	19% (144)	
**Lower poverty line**			
Above LPL	79% (156)	73% (567)	0.071
Below LPL	21% (41)	27% (211)	
**ANC 4**+			
Yes	46% (90)	24% (188)	**< 0.001**
No	54% (107)	76% (590)	
**Received care from SBA**			
Yes	57% (112)	29% (227)	**< 0.001**
No	43% (85)	71% (551)	
**Institutional delivery**			
Yes	53% (105)	24% (187)	**< 0.001**
No	47% (92)	76% (591)	
**Delivery by C-section**			
Yes	15% (29)	9% (70)	**0.018**
No	85% (168)	91% (708)	
**Sex of child**			
Male	46% (90)	52% (407)	0.096
Female	54% (107)	48% (371)	

### 3.2. Coverage of FIC

In Table 3, FIC coverage is reported according to background characteristics of members and non-members of the voucher scheme in the study areas. No significant difference was observed in FIC coverage with sex of child, age of mother, SES and delivery by C-section. However, in case of non-members, FIC coverage was higher for mothers with higher level of schooling, those who received at least 4 ANC visits during pregnancy, and those who had institutional deliveries (Table 3).

**Table 3:** Coverage of fully immunized children (FIC) according to background characteristics and MHVS area.

**Characteristics**	**MHVS area**			
	**Member**		**Non-Member**	
	**% of FIC (FIC/ Total no. of children)**	***P*** **-value**	**% of FIC (FIC/ Total no. of children)**	***P*** **-value**
Number	197		778	
**Age of mother**				
< 25 year	93 (98/105)	0.805	82 (211/257)	0.498
25–34 year	92 (81/88)		85 (361/425)	
35+ year	100 (4/4)		86 (83/ 96)	
**Mother's education**				
None	97 (34/35)	0.405	80 (185/231)	0.016
1–5	90 (64/71)		83 (256/308)	
6 and above	93 (85/91)		90 (214/239)	
**SES**				
Lowest	91 (29/32)	0.182	81 (136/168)	0.400
Second	97 (37/38)		85 (158/186)	
Middle	86 (30/35)		84 (128/153)	
Fourth	98 (47/48)		83 (105/127)	
Highest	91 (40/44)		89 (128/144)	
**Lower poverty line**				
Above LPL	93 (145/156)	0.953	85 (484/ 567)	0.142
Below LPL	93 (38/41)		81 (171/211)	
**ANC 4**+				
Yes	93 (84/90)	0.826	91 (171/188)	0.003
No	93 (99/107)		82 (484/590)	
**Received care from SBA**				
Yes	92 (103/112)	0.560	90 (205/227)	0.003
No	94 (80/85)		82 (450/551)	
**Institutional delivery**				
Yes	92 (97/105)	0.765	91 (170/187)	0.004
No	93 (86/92)		82 (485/591)	
**Delivery by C-section**				
Yes	93 (27/29)	0.962	91 (64/70)	0.082
No	93 (156/168)		83 (591/708)	
**Sex of child**				
Male	91 (97/107)	0.182	85 (344/407)	0.791
Female	96 (86/90)		84 (311/371)	
All	93 (183/197)		84 (655/778)	

Table 4 compares the coverage of FIC between members and non-members of MHVS. Children whose mothers enrolled in MHVS had a higher coverage of FIC than the children whose mother were not enrolled. The regression model adjusting for potential confounders (SES, mother's age and education, sex of children, 4+ ANC, receiving care from SBA, institutional delivery). The coverage was 2 folds higher for members compared to non-members of MHVS, and the adjusted OR was 2.03 (1.11–3.71).

**Table 4:** Coverage of fully immunized children (FIC) according to MHVS area.

**MHVS area**	**% of FIC (FIC/ Total no. of children)**	**Crude OR (95% CI)**	**Adjusted OR^a^ (95% CI)**
Number of children	975		
Non-member	84 (655/778)	Ref.	Ref.
Member	93 (183/197)	2.45 (1.38, 4.37)	2.03 (1.11, 3.71)
Total	86 (838/975)		

^a^adjusted for sex, mother's age and education, SES, ANC 4+, place of birth, receiving care from SBA, delivery by C-section and lower poverty line.

## 4. Discussion

### 4.1. Main findings

The key findings of the study indicate that immunization coverage amongst the children of MHVS beneficiaries was higher than the coverage for children whose mothers were not registered in the voucher programme, even though immunization is not a component of this demand side financing scheme.

### 4.2. Consistencies with previous findings

MHVS ensures utilization of safe motherhood practices, through ANCs during pregnancy, institutional delivery/ delivery by SBAs and PNCs after delivery (13), and through these services, mothers acquire knowledge regarding childhood immunization leading to increased child immunization coverage.

A previous publication on this study established that this voucher programme is effective for pregnant women in utilizing health facilities which played a holistic role in establishing a continuum of maternal health care (4). According to this study, poor voucher recipients had higher utilization of services compared to poor non-voucher recipients. A study in India shows that the promotion of continuation in maternal health care approach advocates an upsurge in child immunization (14). Additionally, a systematic review of studies in LMICs reflects that demand-side financing schemes can increase utilization of maternity services (13). In Bangladesh, utilization of ANC, SBA at delivery, and PNC were found positively associated with MHVS at its early phase (15). Moreover, voucher programmes designed for maternal and child health care have a favorable impact on knowledge, attitude and practice (KAP) of health among pregnant women (16). The KAP gained during pregnancy provides a better understanding about the side effects and benefits of immunization of children on time (17) and thus immunization coverage among children gets an indirect push through MHVS programme.

Besides, several studies support the fact that regular visits to health facility during pregnancy can increase the chance of child immunization. Evidence from a study in India (18) showed that children with mothers visited health facilities for ANC services in the previous 12 months had high chances of being fully vaccinated. This supports and adds to the findings in Nigeria (19), Swaziland (20) and the Africa region (21) that ANC attendance showed a significant effect on the child being fully immunized. Some other studies suggest that those mothers who had ≥3 ANCs, and gave birth at health facilities, as well as children who are the first or second born in the family compared with those who are third born, are more likely to have better immunization coverage (22, 23).

Voucher schemes such as the MHVS not only minimize financial barriers but also raise awareness among women regarding different aspects of healthcare (24) that indirectly narrow down the inequity in other key indicators of health outcomes. MHVS provides voucher to the participants as well as reimburses the cost of services by paying cash to health facilities. There is evidence that financial incentives for both demand side and supply side contribute to increased immunization coverage (25, 26). Hence, the mothers who are members of MHVS are more likely to immunize their children.

### 4.3. Interpretation

This study has shown that MHVS has a positive impact on immunization coverage through increased utilization of safe motherhood practices. Some studies would claim that a full understanding of the benefit of immunization and vaccine-preventable diseases among mothers is associated with the achievement of completed childhood immunization coverage (20, 27–30). Therefore, even though increasing immunization is not a focus of the MHVS, the scheme has still had a non-specific positive effect on it.

### 4.4. Policy implication

Although Bangladesh has commendable childhood immunization coverage, certain pockets of the vaccinable population still cannot be reached and the coverage has been stagnant at 86% (1). Furthermore, Gavi, The Vaccine Alliance's target of “no child left unimmunized” is still to be met and herd immunity has also not been achieved by the population. Majority of the children who are missed out from FIC are the children from low SES and those living in hard-to-reach areas Therefore, avenues such as maternal health programmes targeting the poor and those living in remote areas could be used to attain 100% coverage of FIC.

The MHVS was initiated to improve the health of mothers and children from poor communities, and they continue to have a positive impact on this indicator. While evaluating the scheme, we observed that the scheme also has a non-targeted benefit on increasing immunization coverage of children of beneficiaries as well. This indicates that strengthening national and non-governmental programmes on maternal and child health can result in further improvements of the already strong vaccination coverage among children.

The government of Bangladesh's flagship initiative, community clinics (CC), a unique public-private partnership run through community participation, were set up to extend the reach of primary health services to rural people at grassroot levels. Studies have shown that utilization of CC services is low and can be improved through strengthened community engagement using social accountability approaches (31). Results achieved in this study indicate that strengthening the existing community clinic set up, ensuring relevant and skilled human resources, and intensifying community engagement will not only boost service utilization of community clinics, but will also increase immunization coverage of children as well.

### 4.5. Strengths and weaknesses

This was a pilot study conducted to examine the association of MHVS on childhood immunization coverage, which, to the best of our knowledge, has not been explored before. We included the key characteristics of respondents that have major impact on immunization based on previous studies to get a reliable estimate on immunization coverage. However, since the study was conducted in only four sub-districts of the country where the voucher scheme is running, the results may not reflect the national scenario.

We have addressed several key variables such as mother's age, mother's education, SES, poverty line, ANC 4+, birth by SBA, institutional delivery and sex in establishing the association between voucher receipt and increased immunization coverage among their children.

A limitation of the study is that it excluded children who had no vaccination card or could not show a vaccination card during home visits which could have affected our findings.

### 5. Conclusion

The voucher scheme has both direct and indirect impact on the provision and uptake of various components of maternal and child health services. Our study revealed only one of those non-targeted benefits, that is, increased childhood immunization coverage. Further studies exploring other such benefits could be conducted for further improvement of other health and economic indicators. Moreover, -targeted effects of other government health programmes should also be explored for overall improvement in health status of Bangladeshis through a holistic approach.

## Data availability statement

The raw data supporting the conclusions of this article will be made available by the authors, without undue reservation.

## Ethics statement

Ethical Review Committee of International Center of Diarrhoeal Disease Research, Bangladesh provided approval for the study. Informed written consent was obtained from all respondents.

## Author contributions

SMAH and SSM conceived and designed the study and are the guarantors of the study. SMAH and HD analyzed the data and supervised the data analysis. SMAH, AH, and NS wrote the first draft of the manuscript. All authors contributed to the final version of the manuscript and they had full access to all of the data (including statistical reports and tables) in the study and can take responsibility for the integrity of the data and the accuracy of the data analysis.
