# Supplementary figures and images for: Is the maternal health voucher scheme associated with increasing routine immunization coverage? Experience from Bangladesh
Source: Front Public Health. 2023 Feb 2;11:963162. doi: 10.3389/fpubh.2023.963162 (PMC9937056; doi:10.3389/fpubh.2023.963162)

**Supplementary Figure 1.** Maternal Health Voucher Schem (MHVS) Study sites (sub-districts) in Map
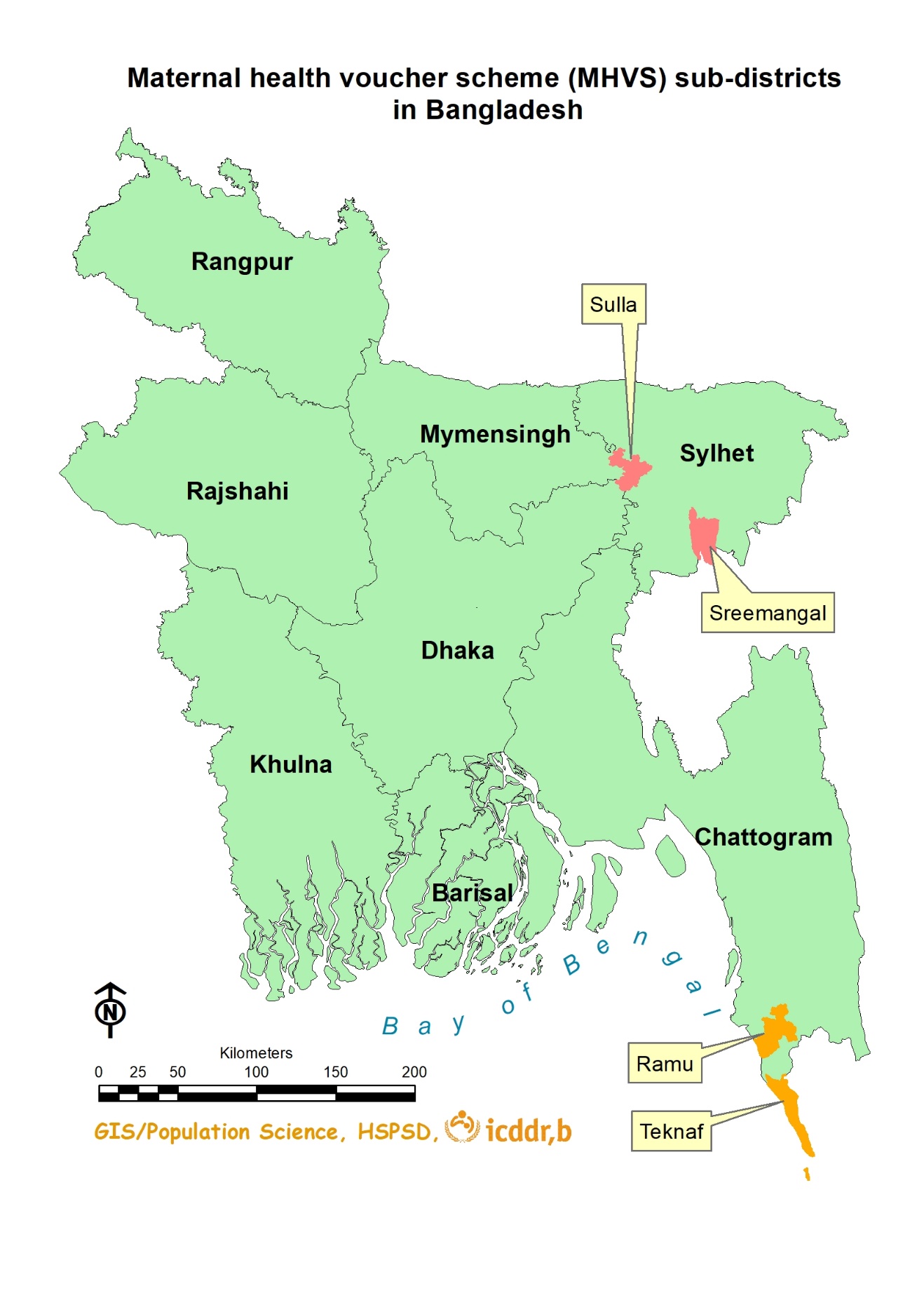

Supplement: Supplementary file 1 [file Table_1.DOCX]
