# Supplementary material for: Is the maternal health voucher scheme associated with increasing routine immunization coverage? Experience from Bangladesh
Source: Front Public Health. 2023 Feb 2;11:963162. doi: 10.3389/fpubh.2023.963162 (PMC9937056; doi:10.3389/fpubh.2023.963162)
Supplement: Supplementary file 2 [file Table_2.DOCX]

**Supplementary Table 1**. Comparison of Maternal, Newborn and Child Health (MNCH) Indicators in Sylhet and Chattogram division with national rates

| **MNCH indicator performance in Chattogram and Sylhet division** | | | |
| --- | --- | --- | --- |
| **Indicators** | **Chattogram (%)** | **Sylhet (%)** | **National (%)** |
| Received at least 4 ANC | 36.3 | 31.8 | 43.7 |
| Assistance at delivery by qualified doctor | 50.2 | 39.3 | 52.7 |
| Facility based delivery | 46.1 | 38.4 | 49.6 |
| Coverage of fully immunized children (FIC) | 83.3 | 61.1 | 83.8 |
| PNC | 50.2 | 40.9 | 52.2 |
